# Tolerance of the Tissues to Foreign Bodies, with Special Reference to the Pulp and Gums

**Published:** 1903-08

**Authors:** M. H. Fletcher

**Affiliations:** Cincinnati, Ohio


### TOLERANCE OF THE TISSUES TO FOREIGN BODIES, WITH SPECIAL REFERENCE TO THE PULP AND GUMS.[1]

BY M. H. FLETCHER, M.D., D.D.S., CINCINNATI, OHIO.

The reason that one foreign substance within the tissues is less or more irritating than another is to be determined not alone by the character of the substance, but also by how it influences the cells of the tissues involved.

---

[1] Abstract of a paper read before the American Medical Association, Section on Stomatology, New Orleans, May 5 to 8, 1903.

That environment influences cell life is well known. Aside from their food, they may be influenced by temperature, electrically, by chemicals, and mechanically. Our present purpose is to consider mechanical irritants.

In treatises on "gunshot wounds" it is presumed that the bullet is of lead, and the practice almost universally advised is to leave the ball undisturbed if it is not easily found. There is an adage generally adhered to, to the effect that "when a bullet has ceased to move it has ceased to do harm."

The accompanying specimens of elephant's tusks, one with a lead bullet encapsulated, the other with an iron bullet rolling about in what seems to have been a pus cavity, shows the different effects these two metals may have on living tissue (assuming the condition to have been the same).

These bullets, which have been shot at the animal's head, have penetrated the pulp-chamber of the tusk, the animal living long enough afterwards for the bullet to become encapsulated in ivory, or produce a pathological cavity.

The protrusion of a broken steel broach from the apex of the root of a pulpless tooth seems invariably to cause an abscess, whereas the protrusion of a gutta-percha point apparently causes no trouble.

A number of years ago Gunn suggested the use of beeswax for packing wounds in place of other dressings, and it is found to be a good procedure, the wax being unirritating to granular surfaces.

Four years ago Gersuny, of Vienna, demonstrated the practicability of injecting even large quantities of paraffin and vaseline into the tissues, for therapeutic as well as for prosthetic purposes.

The writer has been using softened gutta-percha as a therapeutic measure in bone cavities for a number of years. My attention was first drawn to this feature seventeen years ago by having inadvertently injected too much chloropercha (gutta-percha dissolved in chloroform) through a pulpless tooth into a blind abscess. A tumor of this material the size of a pea could be felt at the apex of the tooth, and, since it caused no inflammation and produced no trouble, it was allowed to remain undisturbed, and it is there to-day, never having caused the least disturbance. I believe it is the practice of many dental surgeons to inject this dissolved material through pulpless teeth until it appears at the fistulous opening, expecting the tissues to heal quicker by this treatment than by any other.

Caseous pus may become encapsulated and remain apparently harmless until finally absorbed.

Trichina in small numbers no doubt could remain indefinitely in the tissues without their presence being known, for they are most beautifully taken care of.

Steel needles may wander through the flesh without pain and without apparent damage. Dr. P. S. Conner is authority for the statement that a knife-blade may be tolerated in the same manner, and that iron balls, fragments of shells, splinters, and, in fact, almost any foreign material, if not accompanied by septic matter, may become encapsulated, but agrees that softer, more yielding substances, which are impervious to moisture, are less irritating.

We will assume, then, that as foreign bodies certain substances are, *per se,* noxious or innoxious in degrees proportionate to their physical properties. Those that are most innoxious are impervious to moisture and more or less yielding in character. Hard, smooth substances like a needle or knife-blade may be tolerated, but if they become oxidized or roughened by chemical action they then seem to produce trouble. Hard, rough substances of any kind seem more irritating than a soft pliable material, providing the latter be impervious to moisture. Pieces of cloth or bits of wood seem especially noxious, for reasons which may be explained.

Foreign bodies may be classified as extrinsic or intrinsic, according to whether they are forced into the body from without, or whether they were part of the body, or produced within.

The present object is to consider the manner in which the animal economy deals with sequestra, calcareous deposits about the teeth, and an irritated pulp.

### THE PULP.

In teeth of persistent growth the pulp practically remains one size and is more or less cone-shaped, with its apex directed to the grinding surface and into the long axis of the tooth; the base of the pulp is at the source of supply as shown in the elephant's tusk. When an injury occurs to such a pulp it has every chance of recovery that other tissues have, because of collateral circulation. If the irritant be a foreign body (as shown in the elephant's tusk), it is finally pushed beyond the zone of the pulp, and the formation of dentine proceeds regularly as before. This is true of all tusks and the incisors of all rodents.

In mature teeth of limited growth, as found in man, these conditions are reversed, the apex of the pulp is directed to the apex of the root, and the blood- and nerve-supply is limited to the minimum amount necessary to the life of the pulp, so that in case of injury there is no collateral circulation or nerve-supply for purposes of rapid repair, such as may be found in other tissues; hence the great number of pulpless teeth in human beings, where lesions of the pulp far outnumber those of any other animal.

In filling teeth a pulp may be successfully capped with any suitable innoxious substance, providing the capping does not press the pulp. The walls in which this organ is confined being unyielding, any pressure beyond normal produces inflammation, which cannot be taken care of as it could in other tissues; this is from lack of ability to expand, as is necessary in inflammation and the consequent interference with the circulation, hence the death of the pulp in a comparatively short time. An exposure of the human pulp or other injury to it is most prone to its ultimate death, no matter how successfully the capping may have been done, and it would seem that this proneness to die is due to the very limited supply of vessels. Aside from the difference in mechanical surroundings, there seems no reason why this organ should not recover from injury as easily as any other composed of like tissues, for the process of repair here is no different from that of repair in other tissues of mesoblastic origin. The fact seems to be that the process of repair is practically identical in all tissues, the great essential being abundant blood-supply.

Decay, fillings, cappings, or abrasions may be looked upon as foreign bodies, for they stimulate the pulp to action, resulting in an effort on the part of this organ to protect itself or repair damage done.

The peripheral ends of the fibril may be considered as a part of the pulp, and wearing away or destruction of any part of these delicate processes is a lesion of tissue as truly as an amputation or other larger injuries. Irritation to the pulp, however, which is short of acute inflammation and death usually results in the production of more dentine; this may take the form of nodules, or a protective growth at the pulp ends of the fibril irritated. These growths are shown in the accompanying tooth sections. This effort at protection, however, often results in death from lack of space in which to work, accompanied with the lack of collateral supplies. Compare these results with that of injury to the tusk. Repair in the latter case is not hindered by unyielding confinement as it is in the former, and restoration is complete. Pulp-nodules, in many instances, act as foreign bodies and end with a destructive result to the soft tissue involved. Adherent new growths in the pulp-chamber also have similar endings in many cases, the attempt at repair itself defeating its own object.

Charles B. Nancrede says, "Repair is effected by the same processes in the hard and soft, the vascular and the avascular tissues, the differences being temporary, non-essential, and chiefly dependent upon physical conditions. Thus, the lime-salts render the bone so dense that until they are removed only a limited accumulation of leucocytes and, later, proliferated tissue-cells can take place at the site of injury; yet, from the outset, the soft parts of the bone undergo the same changes, in kind, as does the least compact connective tissue.

"Two forms of repair are usually described, but in reality there is but one, the second variety being, at the outset, only a modification of the first, caused by disturbing influences; when these cease to be operative the processes of repair tend to proceed as at their inception, any variations from the typical methods being accidental, not essential parts of the process. In the normal method reparative processes commence from the moment the physical disturbance of the part ceases and the bleeding is checked. Here the minimum of reparative material is requisite, and the wound is said to heal by the first intention, by simple adhesion, or by aseptic inflammation (obsolete expression), because it is only possible in the absence of infection. Where infection occurs the reparative processes are interfered with and thwarted, reverting eventually to those seen in the absence of suppuration, but vast quantities of reparative materials are wasted, unnecessary tissue-destruction results, and the subsequent changes in the excessively developed germinal tissue often cause serious interference with function. Healing is here said to have taken place by granulation or by second intention, but the end processes are the same in both forms." (Roswell Park, "Treaties on Surgery," p. 350.)

When irritation goes beyond that of the mere stimulation of function, this description of repair seems to cover every field, at least up to our present knowledge, and especially that of pyorrhœa alveolaris.

GUMS AND ALVEOLAR PROCESS.

In the discussion of interstitial gingivitis, so-called pyorrhœa alveolaris, the process of repair, as above described, is the important factor, as it is in all lesions, the exciting cause having been determined.

The local destructive metabolism in this disease is identical with that of any like tissues. Where constant ineffectual efforts are being made to extrude or absorb noxious foreign bodies, we have like results,—namely, destruction of much of the surrounding tissues. In gingivitis the foreign body, in the incipiency of the disease, is nearly always a calcareous deposit.

To Talbot more than to any one else is due our most intimate knowledge of this disease; his extended researches and observation of the disease in man and animals, and his experiments on dogs, bring the matter close under our observation. In his "Conclusions" he says ("Interstitial Gingivitis," p. 148):

"The mass of evidence previously presented demonstrates that the causes of interstitial gingivitis are divisible into predisposing causes (which may be subdivided into local predisposing and constitutional) and exciting causes. The exciting causes are either constitutional or local, but, as a rule, are local or have local action.

"The predisposing factors of this disease, as already mentioned, are conditions of jaw evolution, transitory nature of certain structures, degeneracy, and conditions of previous irritation and inflammation."

In his mercurialization of dogs for the study of the production of the disease by drugs ("Interstitial Gingivitis," p. 117), he says, "Care was taken to secure those in health and with healthy gums."

I should deem it a practical impossibility to know that there was absolutely no deposit about the teeth of these dogs, and think it fair to assume that there must have been some deposits, however small; since domesticated dogs, like men, all have calcareous precipitations about the teeth to some degree, it may not be malignant in its tendency until the etiological moment has arrived, the other factor being lessened resistance by other morbid systemic conditions.

Slight irritation from calcareous deposits may remain *in statu quo* for years, and finally a general morbid condition arrives, or rises to such a pitch that pyorrhœa alveolaris or other advanced conditions of interstitial gingivitis are produced. The inflamed condition of the tissues about the teeth in interstitial gingivitis, it seems, is simply a continual effort at repair, which repair cannot be accomplished on account of the presence of calcareous deposits, or the phagedenic progress of a pus cavity about the roots, the pyorrhœa alveolaris stage. Thus it happens in these cases, as in others, that nature thwarts her own object in her attempt to accomplish it, and destroys much of the surrounding tissue.

The writer's conception of interstitial gingivitis is that it is essentially local in its exciting cause, and that it only has systemic symptoms for the same reasons that other lesions may have auto-intoxication as one result. There seems no reason to believe that drug poisoning or other morbid systemic conditions can produce interstitial gingivitis unless a lesion of the gum pre-exists; this lesion may be the merest break in the mucous membrane, caused by the smallest deposit of this irritating material, this local mechanical irritation being one requisite of the etiological moment. On the other hand, there may frequently be found in gingivitis the systemic disorders accompanying cases of sapræmia and septicæmia.

The continual pressure against the gum tissues of rough irritating calcareous deposits, which continuously increase in quantity and insinuate themselves deeper and deeper beneath the soft tissue, are accompanied with all the products of repair by granulation, or second intention, as described by Nancrede, and may be accompanied with surgical fever. These deposits may be found wherever saliva can penetrate. It has never been the writer's privilege to see deposits of tartar about the necks of the teeth that were innoxious, but they are always irritating to some degree, and usually greatly so. This condition may exist in all stages, from that of being imperceptible to the naked eye up to a complete stage of pyæmia, and may result in death.

In one case which came under my observation death resulted. The culminating cause in this instance was no doubt due to the exposure of more bone by the extraction of a molar while the gums and alveolar process were in the worst possible state. When the patient was presented to me, exfoliation was going on and much bone was exposed, and the patient had a high septic fever. The septic condition was recognized, and advice given against extraction. The patient, however, was suffering greatly with a lower first molar, and went elsewhere to have the tooth removed. I was called again later, but the patient was past recovery, and died within ten days after the extraction.

There is abundant evidence to show that autointoxication, or a low state of health from any cause, greatly favors the progress of the disease, and with this condition present a chronic pus-forming condition may soon be found about one or more of the teeth where the local exciting cause exists, but that autointoxication or other systemic disorders cause this disease, without local irritation, does not appeal to the writer's reason any more than to say that the same disorder causes inflammation of the pleura or conjunctiva without a local point of least resistance from local cause.

Degeneracy or faulty development may bring the etiological moment at a very early stage of the local irritation. This might be almost coincident with the initial lesion, whereas, in normal and healthy individuals, the pyorrhœal stage, even in its mildest form, may be deferred indefinitely, or never appear even where calcareous deposits are excessive.

The fact that the tissues involved are transitory in nature does not seem an adequate factor in accounting for the disease, as suggested by Talbot, since they are as transitory in cases where the disease does not exist as where it does, and these tissues recover as readily as other structures which are not transitory.

There seems no question but that calcareous deposits about the teeth should be looked upon as noxious foreign bodies, and that the constant effort on the part of the tissue to extrude them results in the progressive death of the surrounding tissue. We find in this disease zones of granulation tissue, with the result of destructive metabolism in the soft tissues and the creation of sequestra in the bone. This condition, however, is changed to constructive metabolism the moment the tartar, sequestra, or other local irritants are completely removed.

### SEQUESTRA.

The removal of sequestra by nature is described by Roswell Park as follows:

"A sequestrum may include an entire bone, shaft, or epiphysis, or only a small fragment. A given portion of the bone, having lost its vitality, becomes properly a foreign body which the surrounding tissues endeavor to extrude or to wall off and surround. The extrusive effort is the one which is usually seen. This is done by the continued presence of granulation tissue, which gradually perforates the surrounding bone at places of least resistance, the result being the slow formation of a sinus or several sinuses, ultimately connecting with the surface."

This description, from my point of view, is a very good definition of the pyorrhœal stage of interstitial gingivitis.

So long as the tartar is present as a foreign body the irritation is continuous and sequestra are formed, which are a second source of irritation until they are removed or absorbed.

These cases will all heal by removal of the deposits and sequestra, or by the loss of the tooth. The removal of the teeth invariably results in recovery, and a patient without teeth, either young or old, cannot have the disease, regardless of transitory structures, degeneracy, heredity, drugs, environment, or systemic disease. If lesions of the gums or maxillary bones appear where there are no teeth, it is not interstitial gingivitis, but something else.

